# High signal-to-noise ratio reconstruction of low bit-depth optical coherence tomography using deep learning

**DOI:** 10.1117/1.JBO.25.12.123702

**Published:** 2020-11-15

**Authors:** Qiangjiang Hao, Kang Zhou, Jianlong Yang, Yan Hu, Zhengjie Chai, Yuhui Ma, Gangjun Liu, Yitian Zhao, Shenghua Gao, Jiang Liu

**Affiliations:** a Chinese Academy of Sciences, Cixi Institute of Biomedical Engineering, Ningbo Institute of Materials Technology and Engineering, Ningbo, China; b University of Science and Technology of China, Nano Science and Technology Institute, Suzhou, China; c ShanghaiTech University, School of Information Science and Technology, Shanghai, China; d Southern University of Science and Technology, Department of Computer Science and Engineering, Shenzhen, China; e Shenzhen Bay Laboratory, Shenzhen, China

**Keywords:** optical coherence tomography, image and signal reconstruction, ophthalmic imaging, computational imaging, deep learning

## Abstract

**Significance:** Reducing the bit depth is an effective approach to lower the cost of an optical coherence tomography (OCT) imaging device and increase the transmission efficiency in data acquisition and telemedicine. However, a low bit depth will lead to the degradation of the detection sensitivity, thus reducing the signal-to-noise ratio (SNR) of OCT images.

**Aim:** We propose using deep learning to reconstruct high SNR OCT images from low bit-depth acquisition.

**Approach:** The feasibility of our approach is evaluated by applying this approach to the quantized 3- to 8-bit data from native 12-bit interference fringes. We employ a pixel-to-pixel generative adversarial network (pix2pixGAN) architecture in the low-to-high bit-depth OCT image transition.

**Results:** Extensively, qualitative and quantitative results show our method could significantly improve the SNR of the low bit-depth OCT images. The adopted pix2pixGAN is superior to other possible deep learning and compressed sensing solutions.

**Conclusions:** Our work demonstrates that the proper integration of OCT and deep learning could benefit the development of healthcare in low-resource settings.

## 1 Introduction

Optical coherence tomography (OCT) is a noninvasive cross-sectional high-resolution imaging modality that has been widely used in various medical fields, such as ophthalmology, cardiovascular endoscopy, and dermatology.^1^ In ophthalmology, OCT has become the clinical standard for the examination of nonsuperficial retinal lesions, such as choroidal neovascularization, macular edema, and pigment epithelial detachment.^2^ In the technical aspect, the acquisition speed of OCT systems keeps increasing from tens of hertz at the beginning of its invention to several megahertz today,^3^ which enables the OCT imaging to have fewer motion artifacts, a wider field of view, better resolutions, and higher detection sensitivity.

However, with the evolution of OCT techniques, the increased data size becomes a major issue.^4^ An OCT or functional OCT volume usually has a size of hundreds of megabytes or several gigabytes, which requires not only fast and wide-band analog-to-digital converter (ADC) or frame grabber for data acquisition, but also advanced graphics processing units (GPUs) for real-time imaging alignment. These kinds of hardware significantly increase the cost of OCT device. On the other hand, the large data size influences the transmission efficiency of OCT data among clinical sites, which further impedes the popularization of telemedicine.

To reduce the OCT data size, researchers have tried to first decrease the spatial sampling rate below the Nyquist–Shannon limit and then reconstruct the data using compressed sensing techniques.^5^ Liu and Kang^6^ applied pseudo-random masks to sample part of the CCD pixels and then reconstructed the k-space signal by minimizing the $l_{1}$ norm of a transformed image to enforce sparsity, subject to data consistency constraints. Lebed et al.^7^ proposed a volumetric scan pattern that composed randomly spaced horizontal and vertical B-scans for the reconstruction, then they used this method in the real-time three-dimensional (3-D) imaging of the optic nerve head by a spectral domain (SD) OCT system.^8^ Zhang et al.^9^ employed a k-linear mask to evenly sample the OCT interferogram in the wavenumber domain, which could use less than 20% of the total data and get rid of the spectral calibration and interpolation processes. Fang et al.^10^ reconstructed the low transverse sampled OCT images using sparse representation.

Although the spatial undersampling for the OCT compression has been well explored, there is no attempt to reconstruct the OCT images from a low bit-depth data (undersampling in intensity), to the best of our knowledge. Even though the influences of the bit depth on OCT imaging have been extensively investigated by several groups.^11^^–^^13^ Goldberg et al.^11^ used a swept source (SS) OCT system for human coronary imaging and studied the signal-to-noise ratio (SNR), sensitivity, and dynamic range as a function of the bit depth. They found the SNR increased as the bit depth increased but trended to be stable when the bit depth ≥8. Lu et al.^12^ compared the performance of an 8-bit ADC and a 14-bit ADC in a polarization-sensitive SS OCT system and found the sensitivity and dynamic range dropped due to the low bit depth. Ling and Ellerbee^13^ studied the effects of the low bit depth on the phase of the OCT data and demonstrated that the phase noise could be significantly magnified as the bit depth decreased.

Here, we propose to compress the OCT data by reducing the acquisition bit depth. We further propose to employ emerging deep learning techniques to compensate for the data quality degeneration caused by the low bit depth mentioned above. Deep learning techniques have been successfully used in the data reconstruction of medical imaging modalities, such as magnetic resonance imaging and low-dose x-ray computed tomography.^14^^,^^15^ They also have employed in other types of OCT reconstruction including denoising^16^ and super-resolution.^17^ In low bit-depth data reconstruction, Goi et al.^18^ developed deep-learning-based binary hologram; however, we did not find works related to the low bit-depth reconstruction of OCT data in the literature.

In this paper, we evaluate the feasibility of the proposed idea by converting the low bit-depth OCT images to high bit-depth OCT images using deep learning. The original 12-bit OCT interferogram is quantized into 3- to 8-bit fringes and converted to the OCT images with different bit depths. We employ a pixel-to-pixel generative adversarial network (pix2pixGAN) architecture in the low-to-high bit-depth OCT image transition. The results show it could significantly improve the SNR of the low bit-depth OCT images and are superior to other possible deep learning and compressed sensing solutions. This work demonstrates that the proper integration of low-cost OCT hardware and computational imaging techniques could benefit the development of healthcare in low-resource settings.

## 2 Materials and Methods

### 2.1 Preprocessing

To achieve the lower bit-depth images, we quantize the raw 12-bit interference fringes to simulate sampling depths ranging from 3 to 8 bits with an increment of 1 bit.^19^

**Fig. 1:**
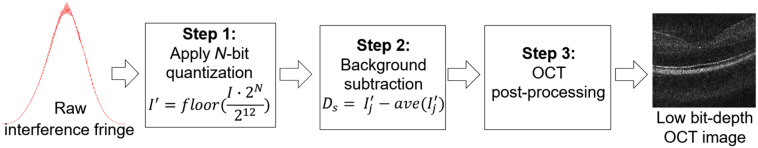
Method to generate the low bit-depth OCT images.

**Fig. 2:**
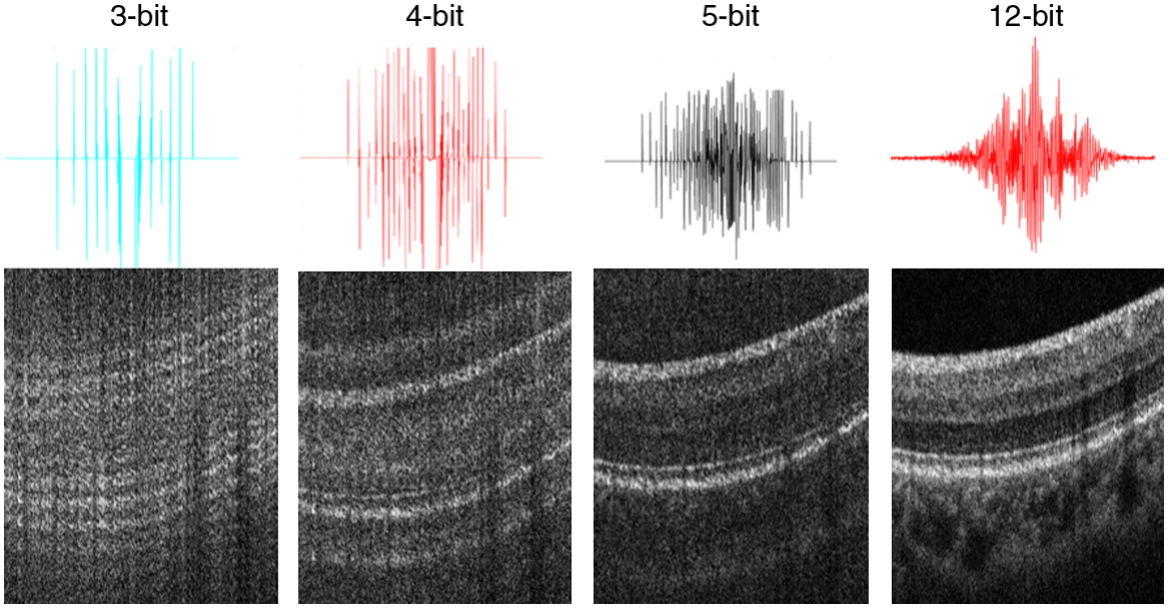
Different bit-depth digital signals correspond to different quality OCT B-scan images.

Figure 1 illustrates the processing steps involved. The original data from the ADC board are read out as the integral values ranging from 0 to $2^{12}-1$. For each bit-depth level, the interference signal’s intensity values are converted from the original 12-bit values using^11^

$$I^{\prime }=floor(\frac{I\cdot 2^{N}}{2^{12}}),$$ (1)

where floor is the floor function that rounds toward negative infinity, N represents the different bit depth, I presents the raw data, while $I^{\prime }$ presents the converted data. Then the background is removed by

$$D_{S}=I_{j}^{\prime }-ave(I_{j}^{\prime }),$$ (2)

where $D_{S}$ presents the signal to remove the background, j presents every column of the data $I^{\prime }$, and function ave calculates the average of each column of the raw matrix. The converted signal is then processed using the OCT postprocessing pipeline, including k-linearization, dispersion compensation, Fourier transformation, and image logarithm. Figure 2 demonstrates different bit-depth digital signals correspond to different quality OCT B-scan images.

### 2.2 Deep Learning Network

In this paper, we propose to use the pix2pixGAN architecture^20^ for the low-to-high bit-depth transition, because the low bit-depth images and high bit-depth ground truths are paired in the pixel level.

**Fig. 3:**
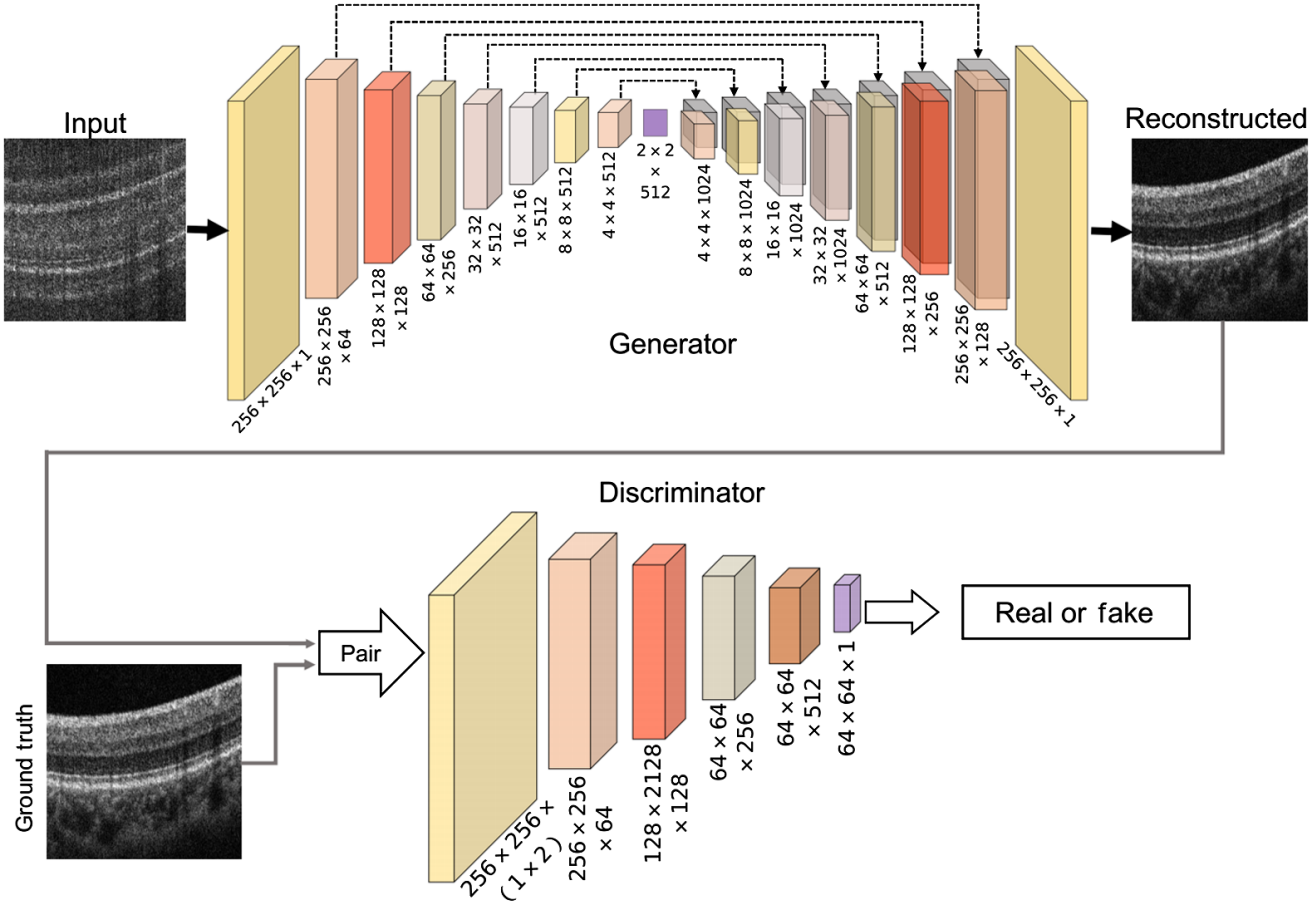
Illustration of the proposed framework for the high SNR reconstruction of the low bit-depth OCT images.

The overall framework is illustrated in Fig. 3. In this framework, the generator is implemented by the U-shape network architecture, which can prevent losing small objects because of the skip connection between each centrally symmetric layer.^21^ As for the discriminator, we adopt the PatchGAN,^20^ which models the OCT image as a Markov random field and only penalizes structure at the scale of patches. The patchGAN restricts the attention of the discriminator to high frequency so that it can avoid blurry results. Using the patches instead of the entire image can reduce the number of parameters and accelerate the training.

The objective of the pix2pixGAN is expressed as^20^

$$L_{GAN}(G,D)=E_{x,y}[\log\;D(x,y)]+E_{x}\{\log[1-D(x,G\{x\})]\},$$ (3)

where the generator G tries to minimize this objective against an adversarial discriminator D that tries to maximize it, x is the input low-bit OCT B-scan image, and y is the corresponding 12-bit depth OCT B-scan set as the ground truth for x. During the training process,^16^ G tries to minimize the goal, while D tries to maximize the goal, so the results are optimized as

$$G^{*}=\arg\;\underset{G}{\min}\;\underset{D}{\max}\;L_{GAN}(G,D),$$ (4)

where $G^{*}$ is the resulted optimized generator.

The purpose of the discriminator remains the same, but the task of the generator is not only to trick the discriminator, but also to approach the ground truth. We use L1 loss instead of L2 to avoid blur

$$L_{L1}(G)=E_{x,y}[{\left\|y-G(x)\right\|}_{1}].$$ (5)

Thus, the final objective is

$$G^{*}=\arg\;\underset{G}{\min}\;\underset{D}{\max}\;L_{GAN}(G,D)+\lambda L_{L1}(G).$$ (6)

### 2.3 Data Preparation and Implementation

With a homemade 70-kHz SD OCT system, we collected 1898 OCT B-scans from a total of 51 subjects (including 13 diseased and 38 normal cases) at a local hospital. We split them on a subject basis for the training, validation, and testing of the SNR reconstruction methods. About 1388 B-scans (including the data from 5 diseased and 34 normal cases) were employed in the training of deep neural networks (DNNs). About 183 B-scans constitute the validation set, which includes the data from 2 diseased and 1 normal cases. We used 327 B-scans in the testing/inference (including 197 B-scans from 6 diseased and 3 normal cases). The human study protocol was approved by the Institutional Review Board and followed the tenets of the Declaration of Helsinki.

The code was implemented in PyTorch and trained on a personal workstation using the NVIDIA GTX 1080ti graphics processing unit (GPU) with 12-GB memory. The operating system is Ubuntu 16.04. To optimize the DNN, we adopt the standard approaches from Ref. 20. We set the epoch=200 and batch size=16. The hyperparameter λ=10. We used the Adam solver^22^ to train the generator from scratch. The initial learning rate was set as $2\times 10^{-4}$. For training the discriminator, we also adopt the Adam optimizer^22^ with the learning rate of $2\times 10^{-4}$. We set $\beta_{1}=0.5$ and $\beta_{2}=0.999$ for both the Adam optimizers. We resized the OCT images to 256×256  pixels for the convenience of the network training. We trained the model from scratch without data argumentation. The model converges at around 100 epochs and costs around 5 hours for each training.

### 2.4 Quantitative Evaluation Metrics

We employ three metrics in the quantitative comparison: peak signal-to-noise ratio (PSNR), multi-scale structural similarity index (MSSSIM), and two-dimensional (2-D) correlation coefficient (CORR2).^23^

The PSNR is an objective standard for evaluating the SNR of an image. It is the ratio between the maximum signal and background noise. The values of the PSNR are in direct proportion to the SNR of an image. It is defined as

$$\mathrm{PSNR}=10\cdot \log_{10}(\frac{{\mathrm{MAX}}_{I}^{2}}{\mathrm{MSE}}),$$ (7)

where the ${\mathrm{MAX}}_{I}$ is the maximum value of the intensity in the OCT images and MSE is the mean squared error.

We employ the MSSSIM to indicate the similarity between two images. Compared with the single-scale structural similarity index, the MSSSIM supplies more flexibility in incorporating the variations of viewing conditions.^24^ The measures of luminance L, contrast C, and structure comparison S are defined as follows:

$$L(X,Y)=\frac{2u_{X}u_{Y}+C_{1}}{u_{X}^{2}+u_{Y}^{2}+C_{1}},$$ (8)

$$C(X,Y)=\frac{2\sigma_{X}\sigma_{Y}+C_{2}}{\sigma_{X}^{2}+\sigma_{Y}^{2}+C_{2}},$$ (9)

$$S(X,Y)=\frac{\sigma_{XY}+C_{3}}{\sigma_{X}\sigma_{Y}+C_{3}},$$ (10)

where X is the tested image and Y is the reference image. $u_{X}$ and $u_{Y}$ are their mean values. $\sigma_{X}$ and $\sigma_{Y}$ are their standard deviations. $C_{1}$, $C_{2}$, and $C_{3}$ are small constants, and here, we take $C_{1}=10^{-4}$, $C_{2}=10^{-4}$, and $C_{3}=0.5C_{2}$.

Thus, the overall MSSSIM evaluation is the combination of these measures at different scales

$$\mathrm{MSSSIM}(X,Y)={\left[L_{M}(X,Y)\right]}^{\alpha_{M}}\overset{M}{\underset{j=1}{\prod }}{\left[C_{j}(X,Y)\right]}^{\beta_{j}}{\left[S_{J}(X,Y)\right]}^{\gamma_{j}}.$$ (11)

The exponents $\alpha_{M}$, $\beta_{j}$, and $\gamma_{j}$ are used to adjust the relative importance of different components. We take M=1, α=1, and β=γ=0.0448.

The CORR2 function implements the Pearson correlation to 2-D arrays^25^ between images A and B. The function is defined as

$$\mathrm{CORR}2=\frac{\underset{m}{\sum }\underset{n}{\sum }(A_{mn}-\overset{¯}{A})(B_{mn}-\overset{¯}{B})}{\sqrt{\left(\underset{m}{\sum }\underset{n}{\sum }{\left(A_{mn}-\overset{¯}{A}\right)}^{2})(\underset{m}{\sum }\underset{n}{\sum }{\left(B_{mn}-\overset{¯}{B}\right)}^{2}\right)}},$$ (12)

where $A_{mn}$ is the intensity of the (m,n) pixel in the image A, $B_{mn}$ is the intensity of the (m,n) pixel in the image B, $\bar{A}$ is the average intensity of the image A, and $\bar{B}$ is the average intensity of the image B.

## 3 Results

### 3.1 Qualitative Evaluation

**Fig. 4:**
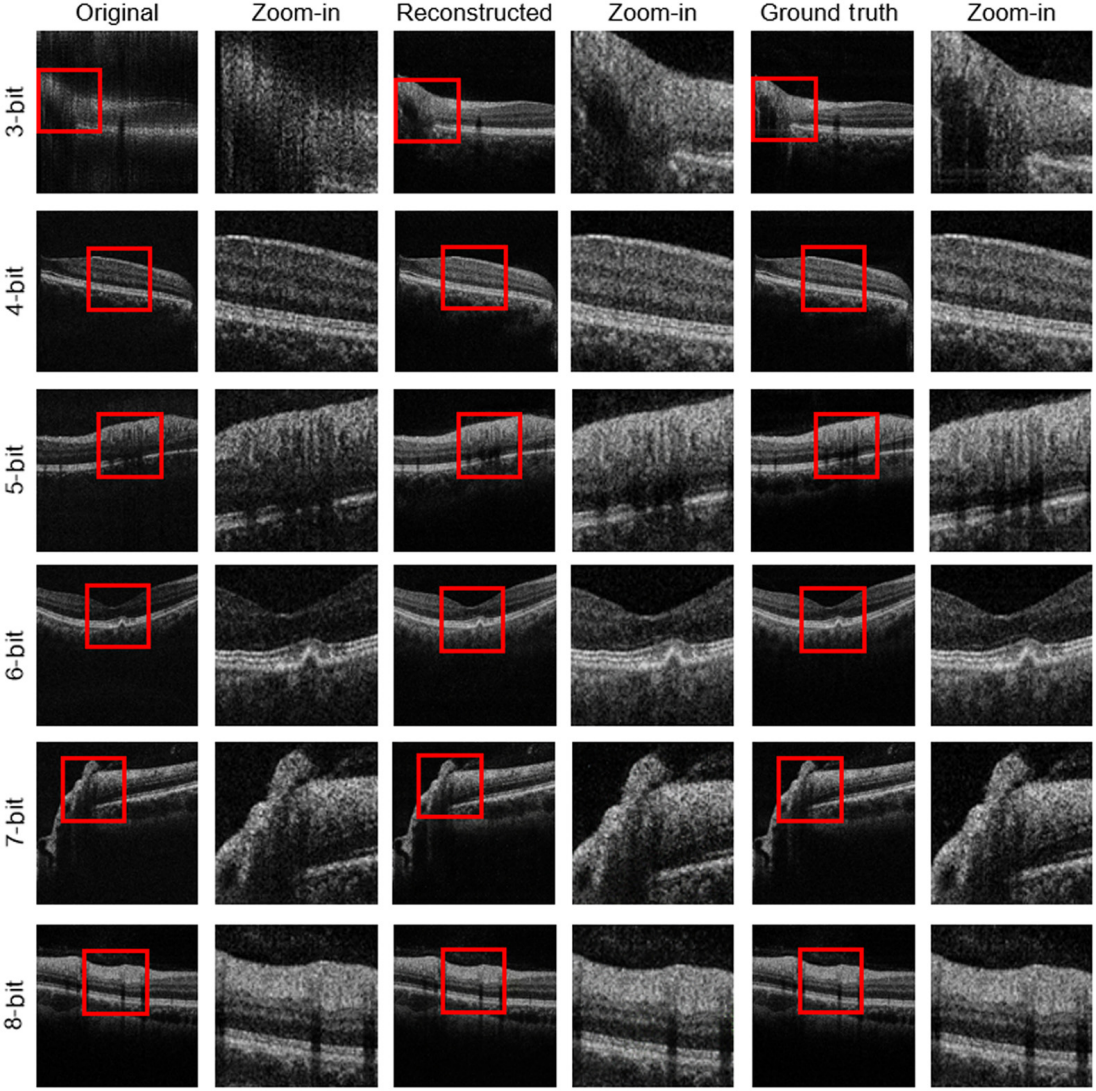
Visual comparison of the original OCT images with different bit depths, their corresponding GAN-reconstructed images, and the enlarged views of the reconstructed images in the red boxes.

Figure 4 demonstrates the visual comparison of the original OCT images with different bit depths, their corresponding GAN-reconstructed images, and the enlarged views of the reconstructed images in the red boxes. The original 3-bit and 4-bit images are quite blurry and are unable to clearly visualize the retinal layers. When the bit depth increases to 5, the blur of the image disappears, but the SNR is very low except for the high reflective inner limiting membrane and retinal pigment epithelium layers. As the bit depth further increases from 6 to 7, the SNR of the image keeps improving, especially the visibility of the choroid. The 8-bit OCT B-scans have good similarity compared with the 12-bit images. The reconstructed images, on the other hand, are presented without the blur and low SNR of the original images even at the 3-bit sampling. All of the reconstructed images have good visibility of the retinal layers and excellent similarity compared with the 12-bit images.

### 3.2 Quantitative Evaluation

We further evaluate this deep-learning-based reconstruction using the quantitative metrics defined in Sec. 2.4. We used the 12-bit images as the reference in the calculation. The PSNR is capable of characterizing the enhancement of the SNR. As shown in Table 1, we can see that the reconstruction can significantly raise the PSNR when the bit depths are low. As the bit depth increases to 8, the reconstruction’s improvement becomes marginal because the original images are very similar to the reference. The MSSSIM and CORR2 are the metrics of similarity from different aspects. As the bit depth increases, these metrics keep rising and getting close to 1. The deep-learning-based reconstruction can significantly improve the similarity of the low bit-depth images. The original high bit-depth images have good similarities, thus the room for improvement becomes low. Even so, the proposed method still can enhance their similarity with the 12-bit reference.

**Table 1:** Comparison of the quantitaitve metrics from the original and reconstructed images with different bit depths.

Bit depth	Image	PSNR (dB)	MSSSIM	CORR2
3-bit	Original	16.351±0.524	0.569±0.012	0.649±0.035
	Reconstructed	21.007±0.331	0.791±0.020	0.899±0.003
4-bit	Original	17.180±0.443	0.636±0.030	0.659±0.028
	Reconstructed	21.452±0.297	0.833±0.014	0.917±0.020
5-bit	Original	19.556±0.343	0.780±0.029	0.817±0.031
	Reconstructed	23.499±0.533	0.854±0.008	0.971±0.008
6-bit	Original	22.297±0.336	0.852±0.020	0.920±0.011
	Reconstructed	25.997±0.505	0.866±0.010	0.971±0.008
7-bit	Original	27.549±0.475	0.953±0.013	0.981±0.023
	Reconstructed	29.501±0.688	0.954±0.007	0.988±0.012
8-bit	Original	33.970±0.457	0.982±0.008	0.996±0.010
	Reconstructed	34.378±0.603	0.980±0.005	0.997±0.003

**Fig. 5:**
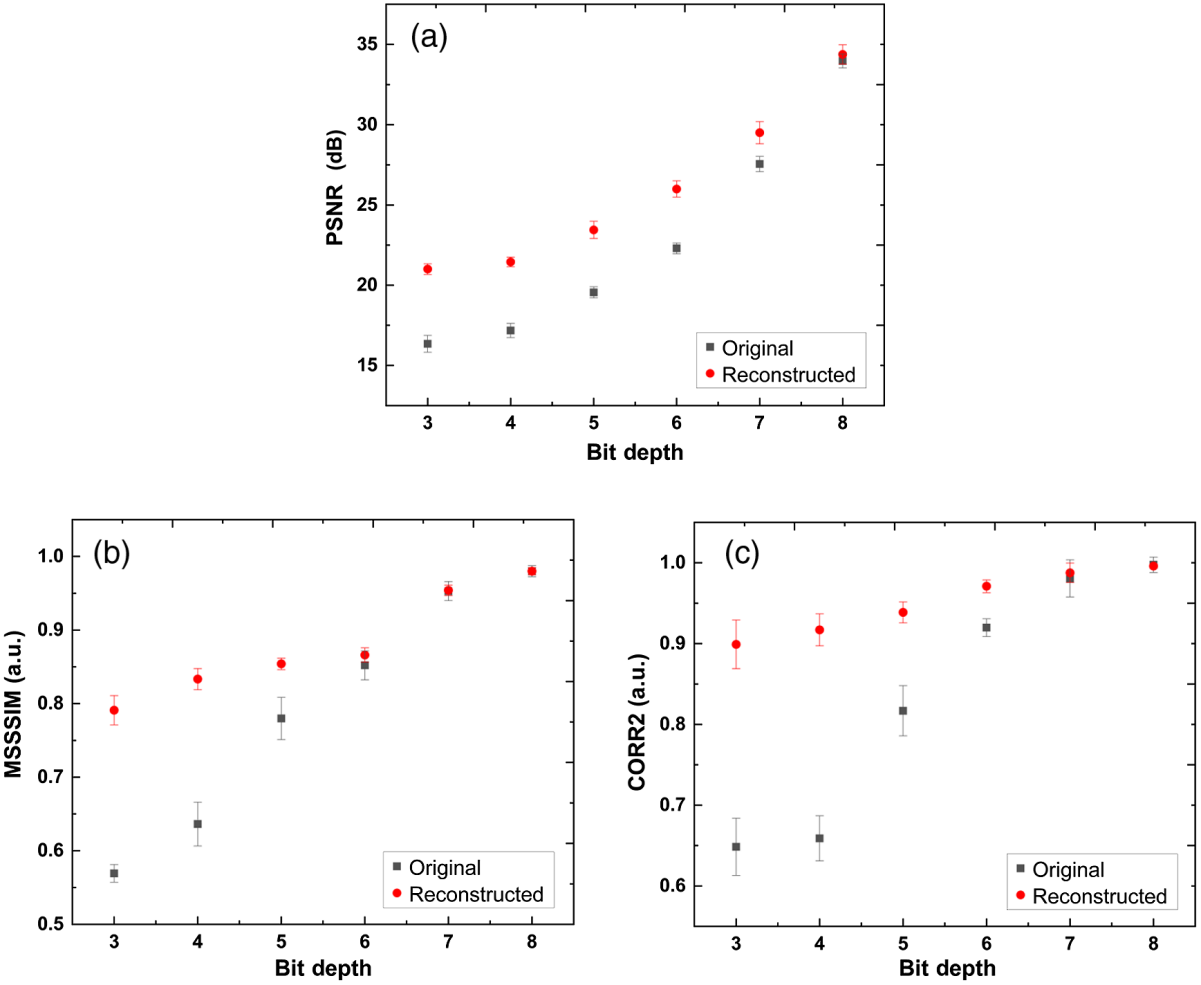
The PSNR (a), MSSSIM (b), and CORR2 (c) as functions of bit depth. The black boxes represent the values of the original images. The red dots represent the values of the reconstructed images.

Figure 5 demonstrates the calculated quantitative metrics as the functions of bit depth. As the bit depth increases, for each metric, the difference of the original and reconstructed images keeps decreasing and converges at the high bit depths. For the two similarity metrics, the MSSSIM and CORR2, there is a leap from the values of the bit depth of 3 to 4 to that of the bit depth of 5, which corresponds to the conversion of the blur OCT B-scans to clear low SNR images as shown in Fig. 4. The metric values of the reconstructed images are always higher than those of the original images, which demonstrates the effectiveness of the proposed approach.

### 3.3 Comparison with Other Deep Learning Methods

Other deep learning methods, such as cycle-consistent G: THAN (cycleGAN),^26^ variational autoencoder (VAE),^27^ and U-shape convolutional network (U-Net),^28^ could also be potentially able to handle this low bit-depth OCT reconstruction task, so we also investigate their performance using the same training and testing configuration as those described in Sec. 2.3.

**Fig. 6:**
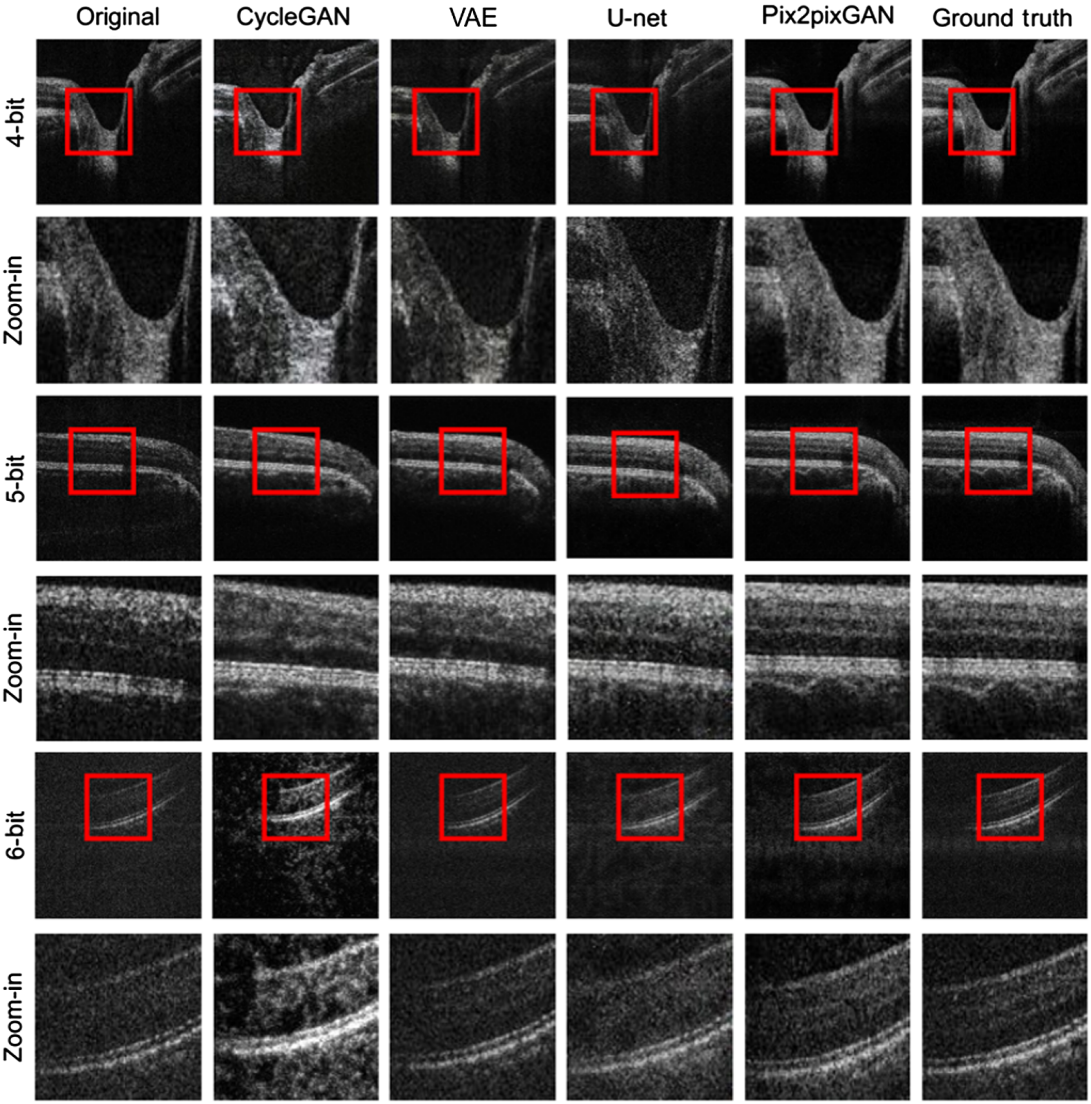
Visual examples of the low bit-depth OCT reconstruction using different deep learning methods. From left to right: original images, the reconstruction results using the cycleGAN, VAE, U-Net, and the pix2pixGAN adopted in this work, and the ground truth. Rows 1, 3, and 5 are the results using 4-bit, 5-bit, and 6-bit images, respectively, and rows 2, 4, and 6 are their corresponding enlarged views inside the red boxes.

Figure 6 shows the representative OCT B-scans processed via these methods and the pix2pixGAN adopted in this work. From left to right are the original images, the reconstruction results using cycleGAN, VAE, U-Net, and the pix2pixGAN adopted in this work, and the ground truth. Rows 1, 3, and 5 are the results using 4-bit, 5-bit, and 6-bit images, respectively, rows 2, 4, and 6 are their corresponding enlarged views inside the red boxes. As demonstrated in the figure, the cycleGAN can reconstruct the OCT images well at the bit depths of 5 and 6, but works poorly at the bit depth of 4. The VAE, on the other hand, is only capable of recovering the global structure of retina but is unable to reconstruct the tissue texture. Different from these two methods, the U-Net and the pix2pixGAN adopted in this work are capable of reconstructing the OCT images at each bit depth. However, the U-Net tends to create a denoising effect on the images and lose the detailed information of blood vessels and their projection shadows.

**Fig. 7:**
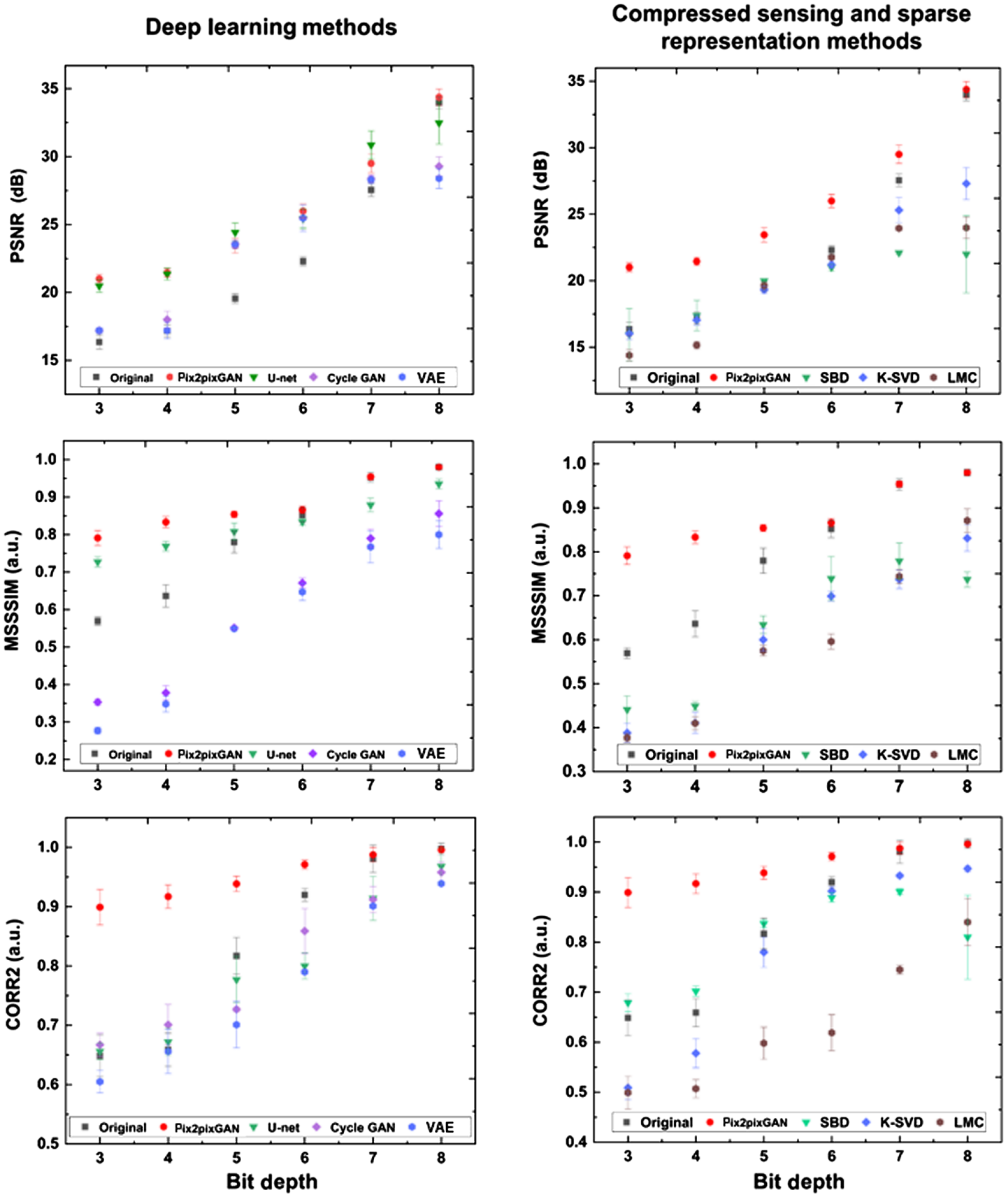
Quantitative metrics of the low bit-depth OCT reconstruction using different methods. From top to bottom are the PSNR, MSSSIM, and CORR2 as functions of the bit depth. The left column is the results using deep learning methods. The right column is the results using compressed sensing and sparse representation methods.

We further calculated the quantitative metrics of the images processed by these methods, as demonstrated in the left column of Fig. 7. From top to bottom are the PSNR, MSSSIM, and CORR2 as functions of the bit depth. In accordance with the visual comparison in Fig. 6, the pix2pixGAN adopted in this work achieves the best performance among different bit depths even though its advantages decrease at higher bit depths because the original images are closer to the ground truth. On the other hand, the U-Net achieves the best PSNR because of its denoising effect, as mentioned before, which does not represent its superiority in this reconstruction task.

### 3.4 Comparison with Compressed Sensing and Sparse Representation Methods

Compressed sensing and sparse representation methods have also been used in the OCT reconstruction, including low-spatial-sampling recovery and speckle noise reduction,^6^^–^^10^^,^^29^^,^^30^ so we also compare the pix2pixGAN adopted in this work with these methods. Specifically, we employ the sparse-based denoising (SBD),^10^ wavelet-based singular value decomposition (K-SVD),^30^ and low-rank matrix completion (LMC)^29^ in this comparison.

**Fig. 8:**
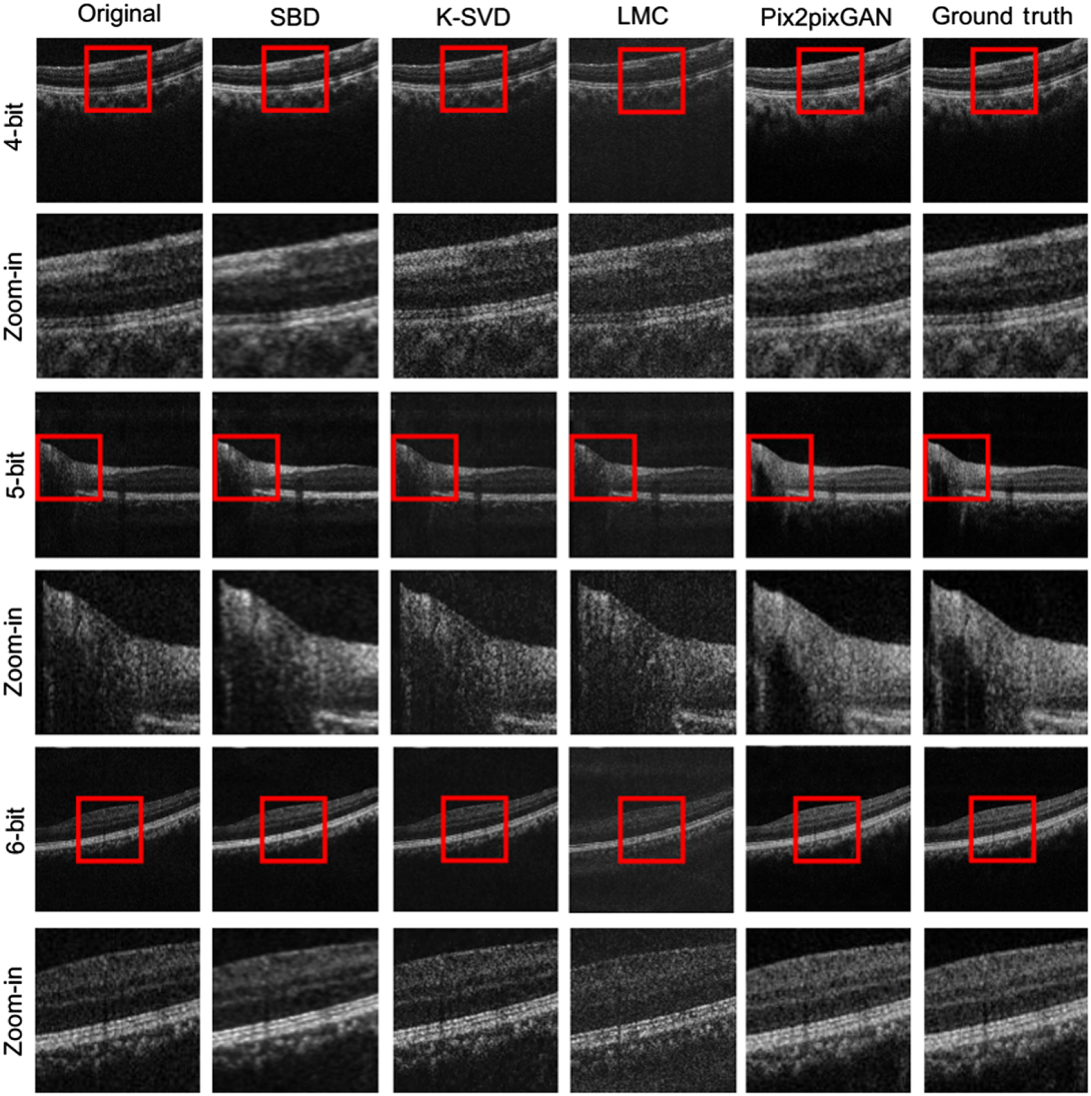
Visual examples of the low bit-depth OCT reconstruction using different sparse representation methods. From left to right: original images, the reconstruction results using the SBD, K-SVD, LMC, and the pix2pixGAN adopted in this work, and the ground truth. Rows 1, 3, and 5 are the results using 4-bit, 5-bit, and 6-bit images, respectively, and rows 2, 4, and 6 are their corresponding enlarged views inside the red boxes.

Figure 8 shows the visual examples of the low bit-depth OCT reconstruction using different compressed sensing methods. From left to right: original images, the reconstruction results using the SBD, K-SVD, LMC, and the pix2pixGAN adopted in this work, and the ground truth. Rows 1, 3, and 5 are the results using 4-bit, 5-bit, and 6-bit images, respectively, and rows 2, 4, and 6 are their corresponding enlarged views inside the red boxes. As shown in the figure, the compressed sensing and sparse representation methods are unable to reconstruct the low bit-depth OCT images properly. Only the enhancement of SNR could be observed in some cases.

The right column of Fig. 7 is the results using compressed sensing and sparse representation methods. In accordance with the visual observation in Fig. 8, the compressed sensing and sparse representation methods have a negative influence on this reconstruction task, while the pix2pixGAN adopted in this work has the best performance for all of the quantitative metrics.

### 3.5 Influence of Deep Learning Parameters

**Fig. 9:**
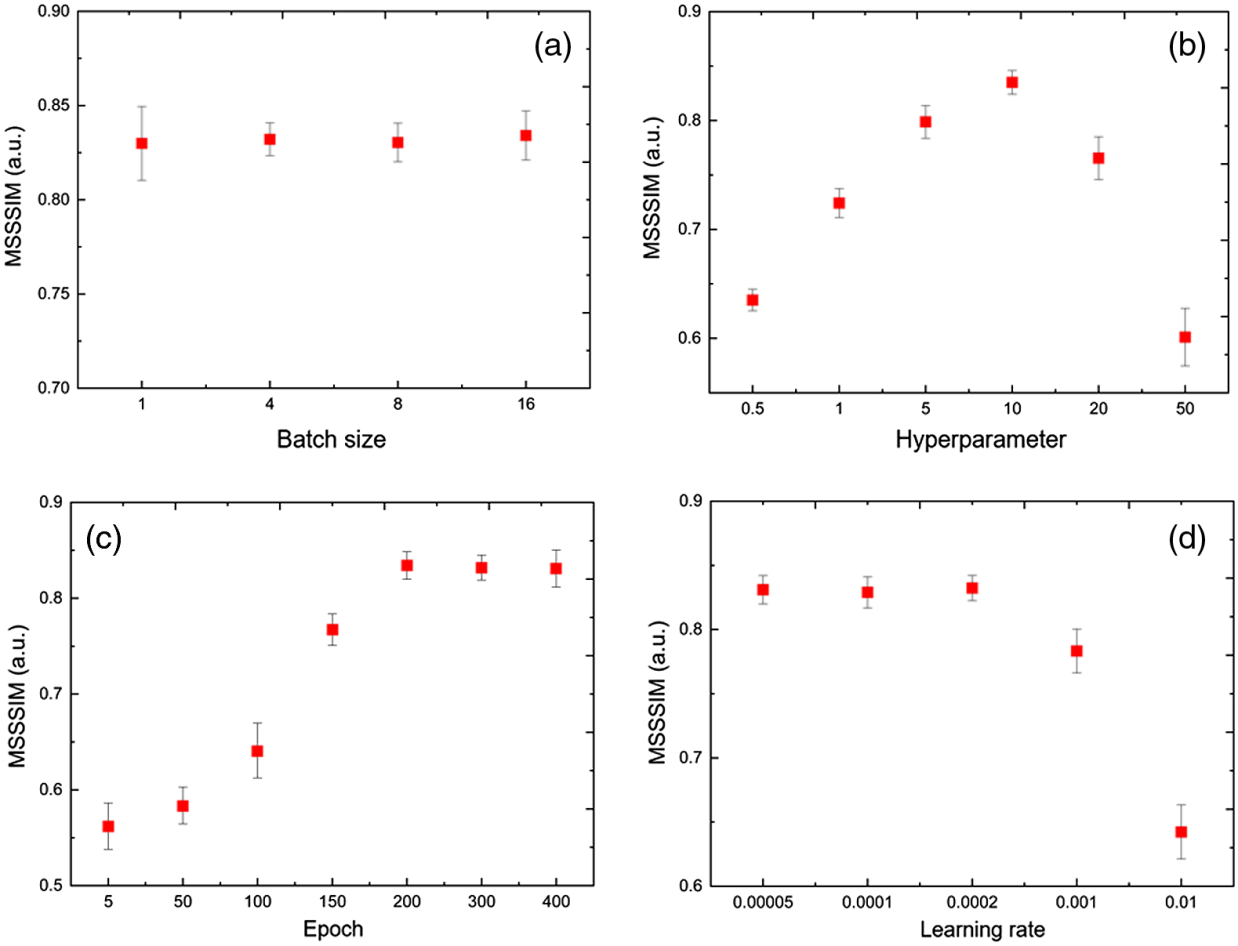
Influence of the (a) batch size, (b) hyperparameter of the L1 loss, (c) epoch number, and (d) learning rate on the results of the deep-learning-based reconstruction. We use the MSSSIM as the quantitative measure, which is derived by comparing the reconstructed image and the 12-bit ground truth.

We further investigate how the selection of the involved deep learning parameters affects the reconstruction results. Figure 9 shows the influence of batch size (a), hyperparameter of the L1 loss (b), epoch number (c), and learning rate (d) on the results of the deep-learning-based reconstruction. We use the MSSSIM as the quantitative measure, which is derived by comparing the reconstructed image with the 12-bit ground truth. We conduct the reconstruction of 4-bit OCT images here.

We can see that the variation of batch size has minimal influence on the MSSSIM. The larger batch sizes result in faster progress in training, but the performance of the trained model seems irrelevant to this parameter after a large number of epochs (e.g., 200). This observation is in consistency with that in Fig. 9(c), where the quantitative measure increases as the number of epochs increase, and tends to be saturated when the epoch >200. We can observe an inverse trend in Fig. 9(d), where the MSSSIM decreases as the learning rate increases. A large learning rate may increase the convergence of training and avoid local minima, but can cause the oscillation of gradient descent, thus miss the global minima. Different from the curves in Figs. 9(a), 9(c), and 9(d), we can see an optimal hyperparameter of the L1 loss at ∼10 in Fig. 9(b), which is in accordance with the observation in the original pix2pixGAN paper:^20^ a large L1 loss would cause blurring while a small L1 would bring artifacts.

**Fig. 10:**
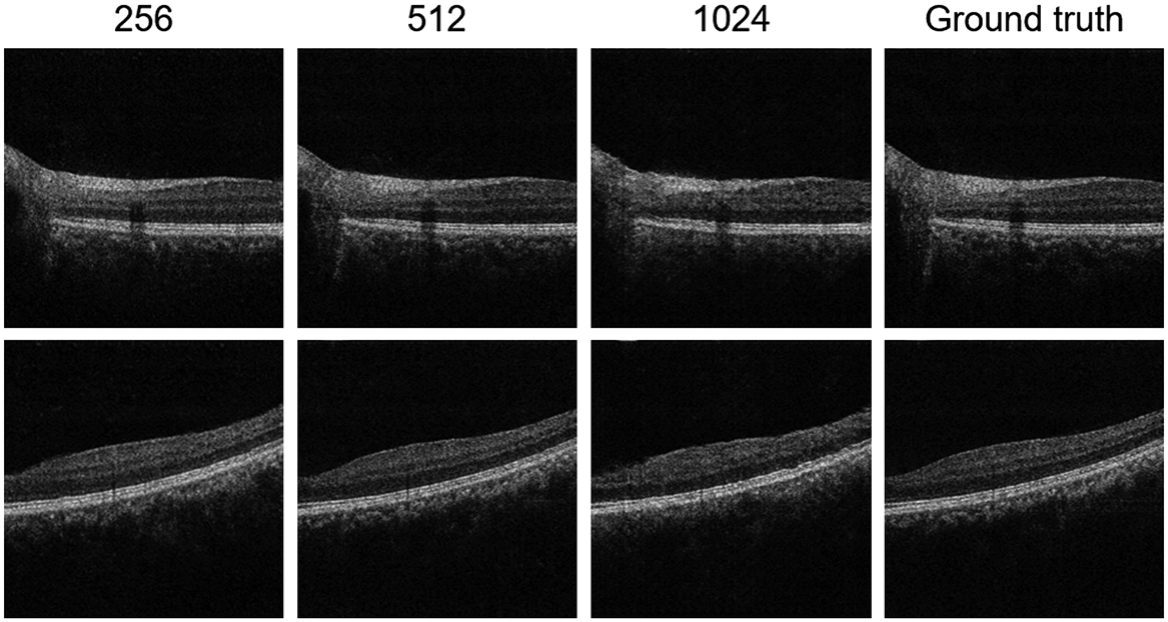
Reconstruction results of using different digital resolutions. From left to right: 256×256  pixels, 512×512  pixels, 1024×1024  pixels, and the ground truth. We also use the 4-bit OCT images for the reconstruction here.

Figure 10 demonstrates the reconstruction results of using different digital resolutions. From left to right: 256×256  pixels, 512×512  pixels, 1024×1024  pixels, and the ground truth. The ground truth OCT images have a resolution of 512×1024  pixels, and we resized them to different digital resolutions using the *imresize* function in MATLAB^®^ 2018a with default parameters. We also use the 4-bit OCT images for the reconstruction here. We can see the influence on image quality is minimal when the resolution increases from 256×256 to 512×512  pixels. However, when the resolution further increases to 1024×1024  pixels, we can see that some details of the reconstructed images are lost, which may be related to the mode collapse problem in GAN-based generation.^31^ To improve the reconstruction at such high resolutions, the deep learning architecture should be redesigned.^32^

### 3.6 Application in Choroid Segmentation

To justify the improvement of the SNR and similarity brought by this deep-learning-based reconstruction method, we employed a graph-search-based algorithm^33^ to segment the choroid-sclera interface (CSI) from the original and reconstructed OCT B-scan with different bit depths. The segmentation of the CSI is the most challenging task among the retinal layers because the OCT probe light is severely attenuated before reaching this layer. Also, the choroid is a vascular layer thus the boundary is composed of large vessels instead of the membranes separating other retinal layers. The light attenuation and the vascular boundary make the CSI very fuzzy. In addition, as mentioned above, the low bit depth would cause the reduction of the SNR, especially at the choroidal region. If the reconstruction succeeds, the SNR of the images will be improved, which further leads to the accurate segmentation of the CSI.

**Fig. 11:**
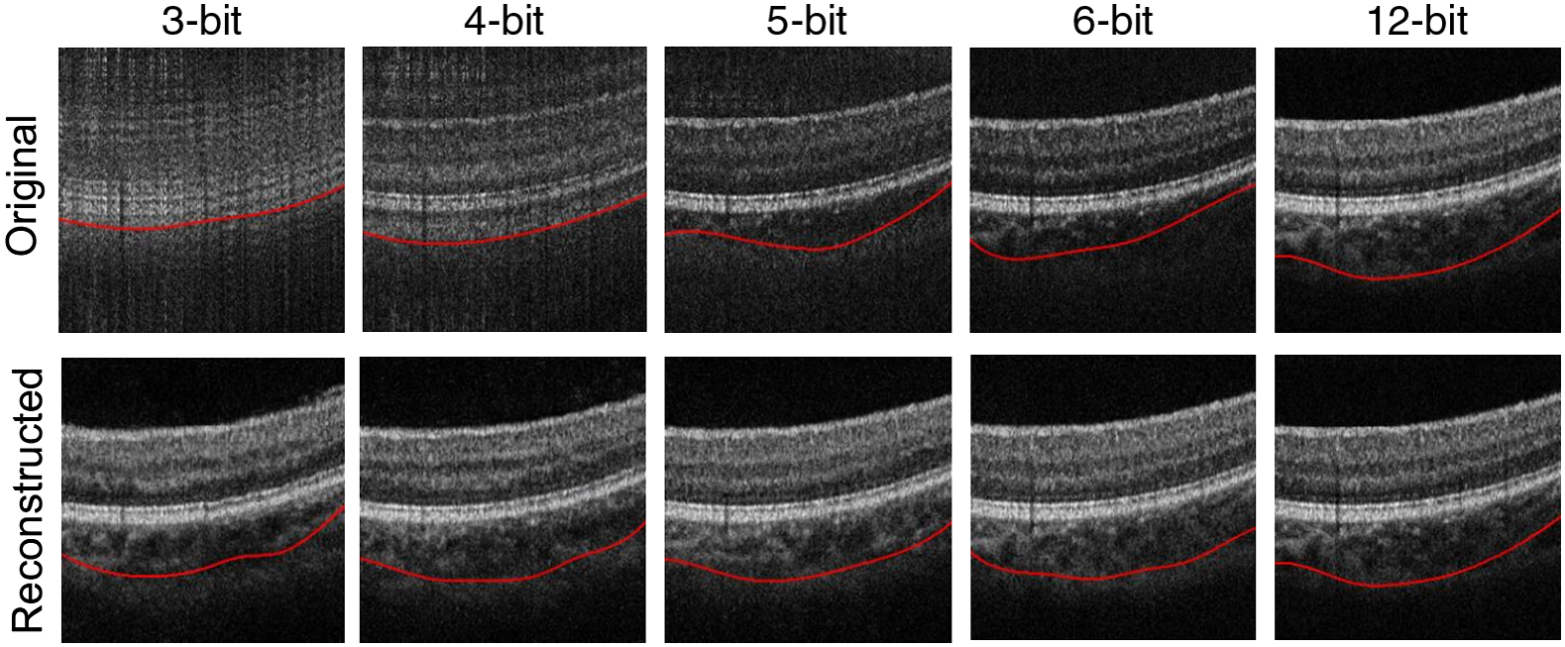
The segmentation results of the CSI (red lines) using the original and reconstructed images with different bit depths.

Figure 11 shows the representative segmentation results of the CSI using different bit-depth B-scans. The red lines indicate the positions of the segmented CSI. We can see the segmentation is very inaccurate in the original low bit-depth images because of the blur or low SNR. After the GAN-based reconstruction, the segmentation is significantly improved because the CSI can be clearly visualized in each image. As the bit depth increases, the segmentation is closer to that of the 12-bit image.

**Fig. 12:**
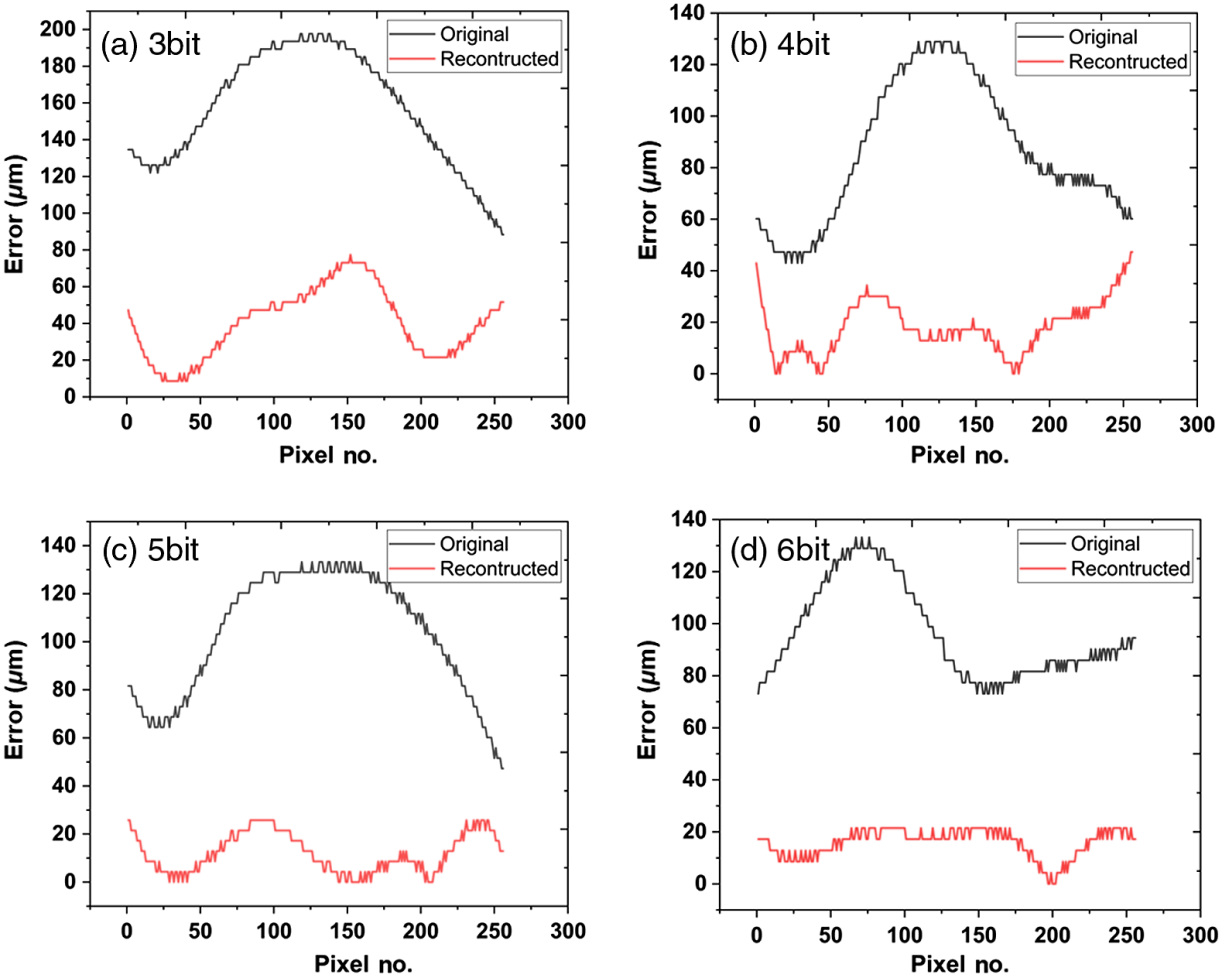
The segmentation errors of the original (black) and reconstructed (red) images with the bit depths of 3- to 6-bit. (a) 3 bit, (b) 4 bit, (c) 5 bit, and (d) 6 bit.

We set the automatically segmented and manually checked CSI of the 12-bit B-scan as the ground truth. Using it as the reference, the segmentation errors of the original and reconstructed images were plotted in Fig. 12. For each bit depth, the errors are significantly decreased using the reconstructed image compared with the errors of the original image. For the reconstructed images, the average segmentation error decreases as the bit depth increases from 39.86  μm at 3-bit to 18.13  μm at 6-bit.

## 4 Discussion and Conclusions

In this paper, we have investigated the feasibility our idea of using an adversarial network to reconstruct the high SNR OCT images using their low bit-depth counterparts. Using the native 12-bit OCT images as the reference, we have found that the GAN-reconstructed images could achieve excellent structure and texture similarity, especially when the bit depth of the original images is ≥5.

**Fig. 13:**
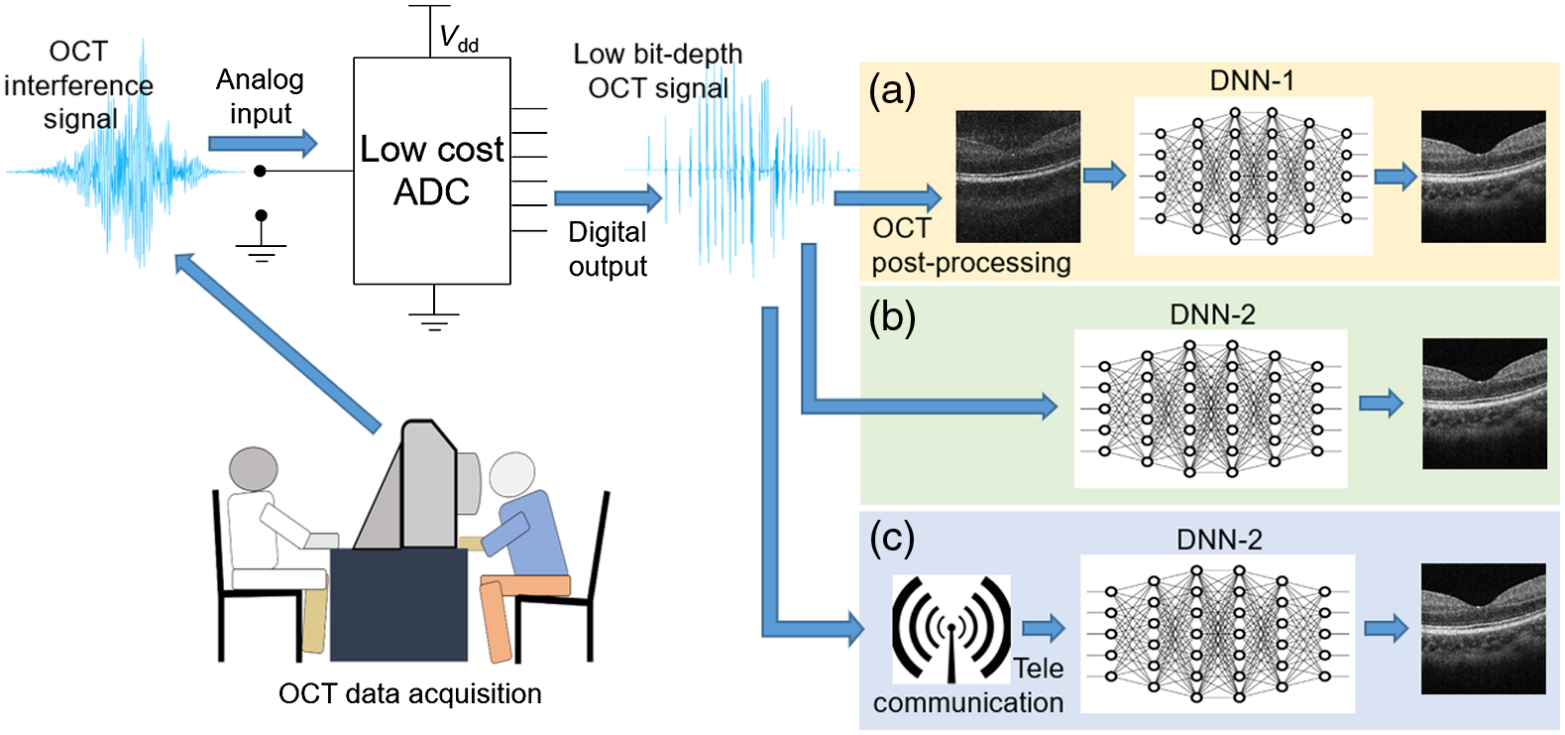
Schematic of reconstructing high SNR OCT images from low bit-depth signals using deep learning. (a) Converting the low bit-depth signals into OCT images and then using a deep neural network (DNN-1) to generate high SNR OCT images. (b) Directly converting the low bit-depth signals into high SNR images using the DNN-2. (c) Using telecommunication to transmit the low bit-depth interferograms to the servers of medical experts then converting them into OCT images using the DNN-2.

The ultimate goal of this study is using the high SNR reconstruction of the low bit-depth OCT to benefit the popularization of this technique (reduce its cost) and the telemedicine, as shown in Fig. 13(c). The previous step of this goal is to convert the original low bit-depth interference signals to the high SNR OCT images through a DNN, as shown in Fig. 13(b). Figure 13(a) gives an alternative approach for OCT data compression in telemedicine as suggested by Mousavi et al.,^34^ which converts the interference signals into OCT images before feeding them into the DNN. Serial numbers 1 and 2 are used to differentiate the DNN used in the image-to-image conversion from the DNN used in the interferogram to image conversion.

The success of this high SNR reconstruction of the low bit-depth OCT images suggests the implementation of the proposed idea illustrated in Fig. 13 can be safely moved to the next stage: the reconstruction from low bit-depth interference fringes to high SNR OCT images. In this reconstruction from OCT raw photoelectric signals to B-scan images, we need to train the DNNs to learn not only the features of different bit depths, but also the characteristics of the entire OCT post-processing including background subtraction, k-linearization, dispersion compensation, Fourier transformation, and image logarithm. Thus, it may not be enough to use the current GAN architecture, so we need to investigate the possibilities of infusing other deep learning architectures, CS techniques, and OCT knowledge for achieving high-quality reconstruction. The progressive realization of the proposed technique will benefit the development of healthcare in low-resource settings and telemedicine.

In summary, we have proposed and implemented a deep learning-based approach to reconstruct the low bit-depth OCT images for achieving high definition and SNR. Since the structure and texture information of the low bit-depth OCT B-scans and the native 12-bit OCT B-scans have precise correspondence, we have adopted the pix2pixGAN architecture in the reconstruction. The GAN-generated images have achieved qualitatively and quantitatively accordance with the native 12-bit OCT images. By comparison with other deep learning and compressed sensing methods, we have validated that the pix2pixGAN is superior in this task. We have further applied the reconstruction images in the segmentation of the CSI and achieved significant improvement in accuracy.

## References

[1] Swanson E. A. Fujimoto J. G., “The ecosystem that powered the translation of OCT from fundamental research to clinical and commercial impact [Invited],” Biomed. Opt. Express 8(3), 1638–1664 (2017). BOEICL 2156-7085 10.1364/BOE.8.001638 28663854 PMC5480569

[2] Adhi M. Duker J. S., “Optical coherence tomography—current and future applications,” Curr. Opin. Ophthalmol. 24(3), 213 (2013). COOTEF 10.1097/ICU.0b013e32835f8bf8 23429598 PMC3758124

[3] Klein T. Huber R., “High-speed OCT light sources and systems [Invited],” Biomed. Opt. Express 8(2), 828–859 (2017). BOEICL 2156-7085 10.1364/BOE.8.000828 28270988 PMC5330584

[4] Yanagihara R. T. et al., “Methodological challenges of deep learning in optical coherence tomography for retinal diseases: a review,” Transl. Vision Sci. Technol. 9(2), 11 (2020). 10.1167/tvst.9.2.11 PMC7347025 32704417

[5] Graff C. G. Sidky E. Y., “Compressive sensing in medical imaging,” Appl. Opt. 54(8), C23 (2015). APOPAI 0003-6935 10.1364/AO.54.000C23 25968400 PMC4669980

[6] Liu X. Kang J. U., “Compressive SD-OCT: the application of compressed sensing in spectral domain optical coherence tomography,” Opt. Express 18(21), 22010–22019 (2010). OPEXFF 1094-4087 10.1364/OE.18.022010 20941102 PMC3001297

[r7] Lebed E. et al., “Rapid volumetric OCT image acquisition using compressive sampling,” Opt. Express 18(20), 21003–21012 (2010). OPEXFF 1094-4087 10.1364/OE.18.021003 20940995

[r8] Young M. et al., “Real-time high-speed volumetric imaging using compressive sampling optical coherence tomography,” Biomed. Opt. Express 2(9), 2690–2697 (2011). BOEICL 2156-7085 10.1364/BOE.2.002690 21991557 PMC3184877

[r9] Zhang N. et al., “Compressed sensing with linear-in-wavenumber sampling in spectral-domain optical coherence tomography,” Opt. Lett. 37(15), 3075–3077 (2012). OPLEDP 0146-9592 10.1364/OL.37.003075 22859090

[10] Fang L. et al., “Fast acquisition and reconstruction of optical coherence tomography images via sparse representation,” IEEE Trans. Med. Imaging 32(11), 2034–2049 (2013). ITMID4 0278-0062 10.1109/TMI.2013.2271904 23846467 PMC4000559

[11] Goldberg B. D. et al., “Performance of reduced bit-depth acquisition for optical frequency domain imaging,” Opt. Express 17(19), 16957–16968 (2009). OPEXFF 1094-4087 10.1364/OE.17.016957 19770914 PMC2785457

[r12] Lu Z. Kasaragod D. K. Matcher S. J., “Performance comparison between 8- and 14-bit-depth imaging in polarization-sensitive swept-source optical coherence tomography,” Biomed. Opt. Express 2(4), 794–804 (2011). BOEICL 2156-7085 10.1364/BOE.2.000794 21483604 PMC3072122

[13] Ling W. A. Ellerbee A. K., “Effects of reduced bit-depth on phase data in common-path optical coherence tomography,” Opt. Express 20(14), 6953–6958 (2012). OPEXFF 1094-4087 10.1364/BIOMED.2012.BTu3A.92 22772258

[14] Zhu B. et al., “Image reconstruction by domain-transform manifold learning,” Nature 555(7697), 487–492 (2018). 10.1038/nature25988 29565357

[15] Han Y. Sunwoo L. Ye J. C., “k-Space deep learning for accelerated MRI,” IEEE Trans. Med. Imaging 39(2), 377–386 (2019). 31283473 10.1109/TMI.2019.2927101

[16] Ma Y. et al., “Speckle noise reduction in optical coherence tomography images based on edge-sensitive CGAN,” Biomed. Opt. Express 9(11), 5129–5146 (2018). BOEICL 2156-7085 10.1364/BOE.9.005129 30460118 PMC6238896

[17] Huang Y. et al., “Simultaneous denoising and super-resolution of optical coherence tomography images based on generative adversarial network,” Opt. Express 27(9), 12289–12307 (2019). OPEXFF 1094-4087 10.1364/OE.27.012289 31052772

[18] Goi H. Komuro K. Nomura T., “Deep-learning-based binary hologram,” Appl. Opt. 59(23), 7103–7108 (2020). APOPAI 0003-6935 10.1364/AO.393500 32788806

[19] Ling W. A. Ellerbee A. K., “The effects of reduced bit depth on optical coherence tomography phase data,” Opt. Express 20(14), 15654–15668 (2012). OPEXFF 1094-4087 10.1364/OE.20.015654 22772258

[20] Isola P. et al., “Image-to-image translation with conditional adversarial networks,” in Proc. IEEE Conf. Comput. Vision and Pattern Recognit., pp. 1125–1134 (2017). 10.1109/CVPR.2017.632

[21] Sato M. et al., “Segmentation of cell membrane and nucleus by improving pix2pix,” in 11th Int. Conf. Bio-Inspired Syst. and Signal Process., BIOSIGNALS 2018-Part of 11th Int. Joint Conf. Biomed. Eng. Syst. and Technol., BIOSTEC 2018, SciTePress, pp. 216–220 (2018).

[22] Kingma D. P. Ba J., “Adam: a method for stochastic optimization,” arXiv: Learning (2014).

[23] Hore A. Ziou D., “Image quality metrics: PSNR vs. SSIM,” in 20th Int. Conf. Pattern Recognit., IEEE, pp. 2366–2369 (2010).

[24] Wang Z. Simoncelli E. P. Bovik A. C., “Multiscale structural similarity for image quality assessment,” in Thrity-Seventh Asilomar Conf. Signals, Syst. & Comput., IEEE, Vol. 2, pp. 1398–1402 (2003). 10.1109/ACSSC.2003.1292216

[25] Ramadan Z. M., “Using entropy and 2-D correlation coefficient as measuring indices for impulsive noise reduction techniques,” Int. J. Appl. Eng. Res. 12(21), 11101–11106 (2017).

[26] Zhu J.-Y. et al., “Unpaired image-to-image translation using cycle-consistent adversarial networks,” in Proc. IEEE Int. Conf. Comput. Vision, pp. 2223–2232 (2017). 10.1109/ICCV.2017.244

[27] Hou X. et al., “Deep feature consistent variational autoencoder,” in IEEE Winter Conf. Appl. Comput. Vision, IEEE, pp. 1133–1141 (2017).

[28] Ronneberger O. Fischer P. Brox T., “U-Net: convolutional networks for biomedical image segmentation,” Lect. Notes Comput. Sci. 9351, 234–241 (2015). LNCSD9 0302-9743 10.1007/978-3-319-24574-4_28

[29] Cheng J. et al., “Speckle reduction in 3D optical coherence tomography of retina by A-scan reconstruction,” IEEE Trans. Med. Imaging 35(10), 2270–2279 (2016). ITMID4 0278-0062 10.1109/TMI.2016.2556080 27116734

[30] Kafieh R. Rabbani H. Selesnick I., “Three dimensional data-driven multi scale atomic representation of optical coherence tomography,” IEEE Trans. Med. Imaging 34(5), 1042–1062 (2014). ITMID4 0278-0062 10.1109/TMI.2014.2374354 25934998

[31] Srivastava A. et al., “Veegan: reducing mode collapse in GANS using implicit variational learning,” in Adv. Neural Inf. Process. Syst., pp. 3308–3318 (2017).

[32] Wang T.-C. et al., “High-resolution image synthesis and semantic manipulation with conditional GANS,” in Proc. IEEE Conf. Comput. Vision and Pattern Recognit., pp. 8798–8807 (2018). 10.1109/CVPR.2018.00917

[33] Mazzaferri J. et al., “Open-source algorithm for automatic choroid segmentation of OCT volume reconstructions,” Sci. Rep. 7, 42112 (2017). SRCEC3 2045-2322 10.1038/srep42112 28181546 PMC5299605

[34] Mousavi M. et al., “Telemedicine + OCT: toward design of optimized algorithms for high-quality compressed images,” Proc. SPIE 8936, 893608 (2014). PSISDG 0277-786X 10.1117/12.2038530

